# The total eclipse of bioinformatics: From disruption to convention, and a gentle warning

**DOI:** 10.1371/journal.pcbi.1014287

**Published:** 2026-06-01

**Authors:** Christos A. Ouzounis

**Affiliations:** Biological‌‌ Computation‌‌ & Computational Biology Group, AIIA, School of Informatics, Aristotle University of Thessalonica, Thessalonica, Greece; Johns Hopkins University, UNITED STATES OF AMERICA

## Into the shadow

Bioinformatics once carried a stigma and had to fight for scientific legitimacy. Emerging as a field, it was treated with great excitement but also significant suspicion by most biologists. The notion of computation as a research tool in a domain primarily guided by experiment was considered almost like a heresy. In those early years, I remember that manuscripts using computation to generate biological results were usually dismissed with the standard response “*Where is the experiment?*” by editors or reviewers. Real biology was then all about pipettes and gels, rather than code and analytics. However, over time, the field has gained acceptance as computation, alongside automation, has proven its value in generating meaningful biological insights at scale.

That heated debate is now over. Computation alone can deliver important discoveries for biology, as we have learned over the last couple of decades. You can do biology without experiment. Or, more precisely, by performing computational, not lab, experiments. The point has moved from slightly radical to self-evident. What remains unsettled, at least in my mind, is the name. What does “bioinformatics” mean in a world where all of biology, as most other natural sciences, is driven by computation? Has this term become a bit of a relic? At a deeper, epistemological level, where does biology stand? Is it still merely an experimental science, “supported” by computation, or has it now entered a new phase, where theory and computation dominate and drive most, if not all, experimental designs?

## From data to discovery

Bioinformatics emerged as a strategic response to the genome projects in the 1990s when genetic sequence data were generated at an unprecedented scale for the first time and needed to be analyzed systematically. The first-generation bioinformatics involved sequence alignment, protein structure prediction, databases and standards, comparative genomics, and later integrated into systems biology, marking the beginnings of a new era in biological research.

And yet, despite those breakthroughs and their rapid adoption, the field was often treated as peripheral or support work, as something secondary to the main foundation of biological research. In particular, this view is amplified by the expansion of data handling and software implementations, which often overshadow the pivotal core ideas and concepts of biological computation that drove the science forward. Consequently, institutes, courses, and, importantly, funding agencies carved it out as something separate, a support theme for “serious” life science research. But this has proven to be an illusion. Bioinformatics was never apart from biology; it was simply the new way of conducting biological research and a natural step forward.

Searls reminded us [1] that the roots of bioinformatics were in scientific discovery, and not in service work. It was never just a toolbox, but a reimagining of what biological science could become.

## Computation took over

Today, the notion that bioinformatics can be clearly distinguished from the rest of biology is almost absurd. Fields such as microbiology, drug development, evolutionary biology, ecology, and neuroscience are all rich in smart algorithms, solid pipelines, interesting simulations, established databases, and lately machine learning models. Modern biology has become virtually indistinguishable from any other computational science, requiring deep domain knowledge of biochemistry and genetics, alongside engineering, mathematics, and computing.

That is to say, bioinformatics has been absorbed into mainstream biology and its practices are now firmly integrated. And what of that old name? Has it started sounding slightly outdated or maybe even misleading?

We have seen this happening before with molecular biology, which was once radical and considered as a threat to “traditional” biology. Today, there is very little ‘molecular’ biology, at least as a distinct notion. The same might already be happening to bioinformatics. The use of advanced computation in biological research is now ubiquitous and indispensable, practically invisible. As Hogeweg rightly argued [2], the field was never just about using computers on biological data, but it was about understanding the informational nature of life itself.

## Words that outlived their meaning

Early in my own career, “bioinformatics” referred to specific tasks such as sequence annotation, database curation, and the first genome comparisons. However, life science research has since expanded beyond these initial roles. Today, systems biology, machine learning, data science, bioengineering, lab automation, or hardware architectures are all within the broader scope of biology, which now includes sophisticated and large-scale experimentally derived measurements, resulting in increased volumes of data. In fact, the controversy surrounding the distinction between bioinformatics and computational biology has also lost its lure, as both engineering and scientific challenges, respectively, are addressed by the same persons, teams, or projects.

This shift is visible in careers, funding schemes, and classrooms. Perhaps fewer people identify as “bioinformaticians,” describing their activities, for example as computational immunology or human genetics, rather than the toolkits they use. Universities, rather than relying on stand-alone bioinformatics courses, are increasingly weaving computational biology into life and computer science degrees, for broader integration. The boundaries have dissolved.

## The eclipse is over

Following the title’s metaphor, I offer here a positive resolution to the question that I posed in 2012 [3], arguing that the alleged ‘eclipse’ has accomplished the ultimate mission. The field succeeded so thoroughly that it virtually erased the boundaries that once defined it. Consider a vague analogy with the discipline of statistics: while statistical science remains a robust discipline in its own right with dedicated departments and journals, its methods are so widely utilized that they have become a standard, often unacknowledged, part of nearly every scientific endeavor. Much like statistics, bioinformatics has become ubiquitous in day-to-day research because its tools and practices are simply everywhere. This was perhaps inevitable.

The question now is not whether computation belongs in biology. We had strong, affirmative answers long ago. The real question is what is the actual role of computation *vis-à-vis* experiment and how we can ensure that the next generation of life scientists can think computationally, or just use computational tools.

We can ascertain with confidence, then, that the full absorption of computation into the very fabric of biology is now complete. What looked like an end was actually an eclipse and now that the eclipse is over, bioinformatics, in this sense, has fulfilled its mission. The term may fade, but the practice will be permanent. Biology entered the realm of scientific computation.

## Science, not service

But here is a warning: in many places, especially in Europe and possibly elsewhere, bioinformatics is being relegated to infrastructure, treated as a “service,” “support,” “foundation,” or a mere “utility.” This trend is increasingly visible in institutional policy decisions and through major funding calls (e.g., within European Commission programs) as purely enabling infrastructures, prioritizing data stewardship, open science or software maintenance over investigator-driven research. Budgets for research shrink, forward-looking agendas dry up, creative projects might be dropped. The same tired argument returns: computation is a “tool,” not “real” biology. This precarious policy undermines the integral role of computation in advancing biological research.

This is a profound misunderstanding. Treating bioinformatics as some kind of cloud-based infrastructure service, away from gifted thinkers, seasoned scientists, doctoral students, and frontier researchers, progress is set back by decades. Bioinformatics is not just a service. Bioinformatics *is* biology [4].

## Life as information

And‌‌ now for the next leap. The proposition that bioinformatics is biology leads to an even bolder statement: biology itself is informatics.

Life, as we know it, can be viewed as the storage, processing, transmission, and regulated flow of information. DNA functions as code. Proteins like functional outputs of that code. Biological evolution like a slow but hugely successful as well as amazingly innovative, constrained optimization algorithm. Cells operate as information-processing entities. Even consciousness can be seen as emergent computation, dictating the next stages of the AI revolution.

This apparent metaphor is in fact a new biology. Space and style do not allow extensive citations to the support of this view. Yet, life as computation is a direction for advanced research in both the life and the computer sciences. This New Biology has revealed itself not as a purely experimental but as an information-rich discipline. The science of living systems can be seen as a science of codes, flows, resilience, error-correction, reproduction, adaptation, and optimization. This is what life is, under a new lens.

With the recent advances in Artificial Intelligence, computation takes on a new meaning, where even computationally generated hypotheses can be tested directly by experiment. Those novel processes with minimal human-in-the-loop will further the scope of biological computation at unimaginable speed, efficiency, and sophistication, if properly realized.

## Nothing in biology makes sense…

Bioinformatics was born under suspicion, matured under defiance, and might be fading as a label exactly because it succeeded. Somehow, with twists and turns, it has become a major part of biology. And biology, in turn, can be redefined as an information discipline.

Yes, the eclipse is over: a re-emergence after the disappearance. What some once mistook for the demise of bioinformatics was a victory and a step forward: an inter-disciplinary enterprise into the very heart of modern science.

Paraphrasing Dobzhansky’s famous motto on evolution (*American Zoologist 4(4), Nov 1964*), with a certain conviction, we can now say: *nothing in biology makes sense except in the light of* *computation*. Bioinformatics is the New Biology. Biology is the New Informatics. The eclipse has waned, pointing to a new, brighter beginning.
